# Advanced Powder Metallurgy Technologies

**DOI:** 10.3390/ma13071742

**Published:** 2020-04-08

**Authors:** Pavel Novák

**Affiliations:** University of Chemistry and Technology, Prague, Department of Metals and Corrosion Engineering, Technická 5, 166 28 Prague 6, Czech Republic; panovak@vscht.cz

**Keywords:** powder metallurgy, mechanical alloying, spark plasma sintering, self-propagating high-temperature synthesis

## Abstract

Powder metallurgy is a group of advanced processes for the synthesis, processing, and shaping of various kinds of materials. Initially inspired by ceramics processing, the methodology comprising of the production of a powder and its transformation to a compact solid product has attracted great attention since the end of World War II. At present, there are many technologies for powder production (e.g., gas atomization of the melt, chemical reduction, milling, and mechanical alloying) and its consolidation (e.g., pressing and sintering, hot isostatic pressing, and spark plasma sintering). The most promising ones can achieve an ultra-fine or nano-grained structure of the powder, and preserve it during consolidation. Among these methods, mechanical alloying and spark plasma sintering play a key role. This Special Issue gives special focus to the advancement of mechanical alloying, spark plasma sintering and self-propagating high-temperature synthesis methods, as well as to the role of these processes in the development of new materials.

Powder metallurgy is a relatively old discipline, as some trials date back to at least 3000 B.C. The technological importance of this branch starts at the beginning of the 20th century. To date, it has undergone significant development through the hot isostatic pressing process, which allows the achievement of an isotropic structure, metal injection molding and other methods for net shape production to the methods used to obtain nanostructured materials. Among these methods, mechanical alloying for powder production, and spark plasma sintering for its consolidation, probably play the most important role. These methods are applied for the manufacture of a Ni-Co-Cr-Fe-Ti high entropy alloy by Moravcik et al. [1]. The results of this paper show that Ti increases the strength of the alloys by solid solution strengthening, reaching an ultimate tensile strength of approx. 1600 MPa, together with the ductility of 9 %, even though there are oxide inclusions in the material. The presented results could lead to future, novel inclusion-tolerant materials. Three papers in the Special Issue are focused on the processing of Fe-Al-Si-based materials by the same set of methods [2,3,4]. The Fe-Al-Si alloys exhibit very good oxidation resistance against high-temperature oxidation. As well as the binary Fe-Al alloys and some other aluminide systems, these materials show the anomaly of not only the yield strength, but also of the ultimate tensile strength. This means that these mechanical properties increase with the temperature in some temperature intervals (for these alloys at 400–500 °C). This increase in strength is also coupled with considerable ductility, even though these alloys are brittle at room temperature. Together with oxidation resistance and thermal stability, this implies that these alloys could probably be applied, for example, as exhaust valves of internal combustion engines [2]. The properties of this alloy could be further improved by the addition of a combination of molybdenum with nickel or titanium. This leads to very good tribological properties, leading to a significantly lower wear rate than in the case of high-performance tool steels [3]. Cech et al. investigated how the feedstock powder composition influences the structure and properties of Fe-Al-Si alloys [4]. The authors compare the use of elemental powders (Fe,Al,Si) with pre-alloyed ones (Fe-Al, Fe-Si and Al-Si alloys). There are no significant differences in the phase composition and microstructure of the products, dependending on the feedstock powder composition. However, the use of softer elemental powders shortens the mechanical alloying process. The pre-alloyed Fe-Al powder improved the fracture toughness of the alloy. Ti-Al and Ti-Al-Si alloys are, as well as Fe-Al based alloys, known as high-temperature materials, which are problematically produced by common metallurgy methods. In this Special Issue, the possibility of the synthesis of Ti_3_Al intermetallic phase by self-propagating high-temperature synthesis from elemental titanium and aluminium powder is described [5]. It was found that the reactions between titanium and aluminium powder start with the formation of a metastable Ti_2_Al_5_ phase. This phase then reacts with titanium, leading to the desired Ti_3_Al phase. The series of Ti-Al-Si alloys with varying ratios between titanium, aluminium and silicon was prepared by the combination of mechanical alloying and spark plasma sintering [6]. As a reference, melting metallurgy was applied to alloys of the same chemical composition. The powder metallurgy could obtain a significantly finer microstructure and reach a correspondingly higher ultimate tensile strength. On the other hand, the fracture toughness of the melting-metallurgy-prepared samples was higher.

A significant part of the Special Issue is devoted to the functional materials, which are used for their shape memory properties, magnetic behaviour, optical properties, self-healing beahaviour or biodegradability in the organism. Salvetr et al. studied the synthesis of a NiTi shape memory alloy by self-propagating high-temperature synthesis, milling and spark plasma sintering [7]. The alloy reached almost the theoretical density, a very high ultimate compressive strength of more than 2000 MPa, but it lost the transformation behaviour. Further heat treatment at the temperature of 600–700 °C with slow cooling was needed to recover the transformation behaviour, which is crucial for the shape memory effect [7]. The magnetic materials were investigated by Skotnicova et al. [8]. This paper focused on the coercivity enhancement of Nd-Fe-B magnets by optimizing the microstructure by the addition of a Dy_3_Co_0.6_Cu_0.4_H_x_ alloy during mechanical activation. The whole-powder metallurgy process included strip casting, hydrogen decrepitation, milling (mechanical activation) and sintering. The results show the possibility of using a unified initial alloy for the manufacture of magnets with tailored magnetic characteristics.

Powder metallurgy was initially inspired by ceramics processing, while the spark plasma sintering was originally developed for the processing of metals. Nowadays, this method allows for strong development in the processing of special ceramic materials. In this Special Issue, magnesium aluminate spinels for optical applications, with the addition of LiF, MnF_2_, and CoF_2_ were consolidated this method [9]. Returning back to the world of metals, a composite material composed of aluminium alloy and nickel particles, prepared by a combination of gas atomization ańd spark plasma sintering, is presented [10]. This material reveals the functionality of self-healing, which is relatively new and unusual for metals. Kubasek et al. [11] presents a novel biodegradable composite material composed of zinc and magnesium, which can possibly be applied in future surgery for the temporary fixing implants. The problematics of composite materials are also solved from the viewpoint of the advancement of the mixing process for ternary powder mixtures [12].

Materials for cutting tools usually contain elements such as cobalt and tungsten, which are listed by the European Commission as critical raw materials (CRM). The review [13] shows the ways that these elements can be substituted in the manufacturing sector and recycled to minimize their economic impact, and how to improve the manufacturing process itself. One of the materials which are applicable as tool materials is maraging steel. This substitute for conventional materials is very popular because it can be processed by 3D-printing methods. The selective laser melting process using the atomized powder of the maraging steel was described, as well as the subsequent post-processing heat treatment [14].

As seen above, the Special Issue *Advanced Powder Metallurgy Technologies* provides insight into the recent trends in powder metallurgy from the viewpoints of both the advancement of the technology and the development of new materials using these methods. It is great to see that the modern powder metallurgy can move technology, economics, and also healthcare, forward.
